# Osteocyte Mechanotransduction in Orthodontic Tooth Movement

**DOI:** 10.1007/s11914-023-00826-2

**Published:** 2023-10-04

**Authors:** Hadi Seddiqi, Jenneke Klein-Nulend, Jianfeng Jin

**Affiliations:** Department of Oral Cell Biology, Academic Centre for Dentistry Amsterdam (ACTA), Amsterdam Movement Sciences, University of Amsterdam and Vrije Universiteit Amsterdam, Gustav Mahlerlaan 3004, 1081 LA Amsterdam, The Netherlands

**Keywords:** Bone remodeling, Jaw bone, Mechanical loading, Mechanotransduction, Osteocyte, Orthodontic tooth movement

## Abstract

**Purpose of Review:**

Orthodontic tooth movement is characterized by periodontal tissue responses to mechanical loading, leading to clinically relevant functional adaptation of jaw bone. Since osteocytes are significant in mechanotransduction and orchestrate osteoclast and osteoblast activity, they likely play a central role in orthodontic tooth movement. In this review, we attempt to shed light on the impact and role of osteocyte mechanotransduction during orthodontic tooth movement.

**Recent Findings:**

Mechanically loaded osteocytes produce signaling molecules, e.g., bone morphogenetic proteins, Wnts, prostaglandins, osteopontin, nitric oxide, sclerostin, and RANKL, which modulate the recruitment, differentiation, and activity of osteoblasts and osteoclasts. The major signaling pathways activated by mechanical loading in osteocytes are the wingless-related integration site (Wnt)/β-catenin and RANKL pathways, which are key regulators of bone metabolism. Moreover, osteocytes are capable of orchestrating bone adaptation during orthodontic tooth movement.

**Summary:**

A better understanding of the role of osteocyte mechanotransduction is crucial to advance orthodontic treatment. The optimal force level on the periodontal tissues for orthodontic tooth movement producing an adequate biological response, is debated. This review emphasizes that both mechanoresponses and inflammation are essential for achieving tooth movement clinically. To fully comprehend the role of osteocyte mechanotransduction in orthodontic tooth movement, more knowledge is needed of the biological pathways involved. This will contribute to optimization of orthodontic treatment and enhance patient outcomes.

## Introduction

The demand for orthodontic tooth movement is increasing as the health and expectations of our population improve. Orthodontic treatment allows tooth movement based on the periodontal ligament, a specialized connective tissue attaching the tooth root to the alveolar bone [1]. Orthodontic tooth movement aims to move malpositioned teeth to an appropriate position through periodontium remodeling induced by orthodontic force [2••]. The forces exerted on a tooth result in stress on the periodontal ligament. Consequently bone resorption is induced in areas of compression, and bone formation in areas of traction or stretching, leading to tooth movement [3]. A healthy periodontium is needed to prevent damage to the tissues supporting the tooth [4]. Orthodontic tooth movement uses teeth segments that resist movement and operate as “anchors” to pull against other teeth segments that are intended to be moved [5]. The term ‘anchorage’ in orthodontics is used to describe the resistance to tooth movement resulting from reciprocal forces [6]. Assuming ideal treatment goals, anchorage requirements should be evaluated in all three planes of spaces: anterior–posterior, transverse, and vertical [7]. Among anchorage devices, e.g. miniscrews have increasingly been used for orthodontic anchorage because of their absolute anchorage, easy placement and removal, and low cost [8, 9]. Successful orthodontic treatment outcomes depend on adequate control of anchorage, which is an important limiting factor in orthodontics [10]. Orthodontic anchorage signifies the nature and degree of resistance to displacement offered by an anatomic unit [10]. Depending on the desired treatment outcome, tooth movements should be optimized.

Orthodontic tooth movement depends on coordinated tissue resorption and formation in the adjacent bone and periodontal ligament [11]. Mechanical loading on teeth causes fluid flow, initiating an aseptic inflammatory cascade culminating in osteoclastic bone resorption in compression areas and osteoblastic bone deposition in tension areas [11]. Compression and tension are associated with ascertaining local mechanical loading to regulate bone and periodontal ligament remodeling for tooth displacement [11]. Efficient and safe orthodontic tooth movement relies on the underlying biological and biomechanical mechanisms of orthodontic tooth movement. Throughout life, bone undergoes remodeling, which involves the resorption of mineralized bone by osteoclasts followed by the formation of new mineralized bone by osteoblasts [8, 9]. The remodeling cycle consists of a resorption phase, when osteoclasts digest old bone, a reversal phase, when mononuclear cells appear on the bone surface, and a formation phase, when osteoblasts deposit new bone until the resorbed bone is entirely replaced [12]. The coordinated activity of osteoclasts and osteoblasts during bone adaptation is generally considered to be orchestrated by mechanosensitive osteocytes [13, 14]. Osteocyte morphology (shape) varies in different types of bone, and reflects differences in mechanosensitivity. Round osteocytes are more sensitive to mechanical force than elongated osteocytes [15]. Moreover, osteocytes with different surface area and orientation are existing locally in particular areas of the maxillary bone, which is related to the magnitude and orientation of mechanical force [16•]. The relationship between osteocyte shape and orientation and mechanical force in the jaw bone might contribute to a success of dental implants and orthodontic tooth movement.

**Fig. 1:**
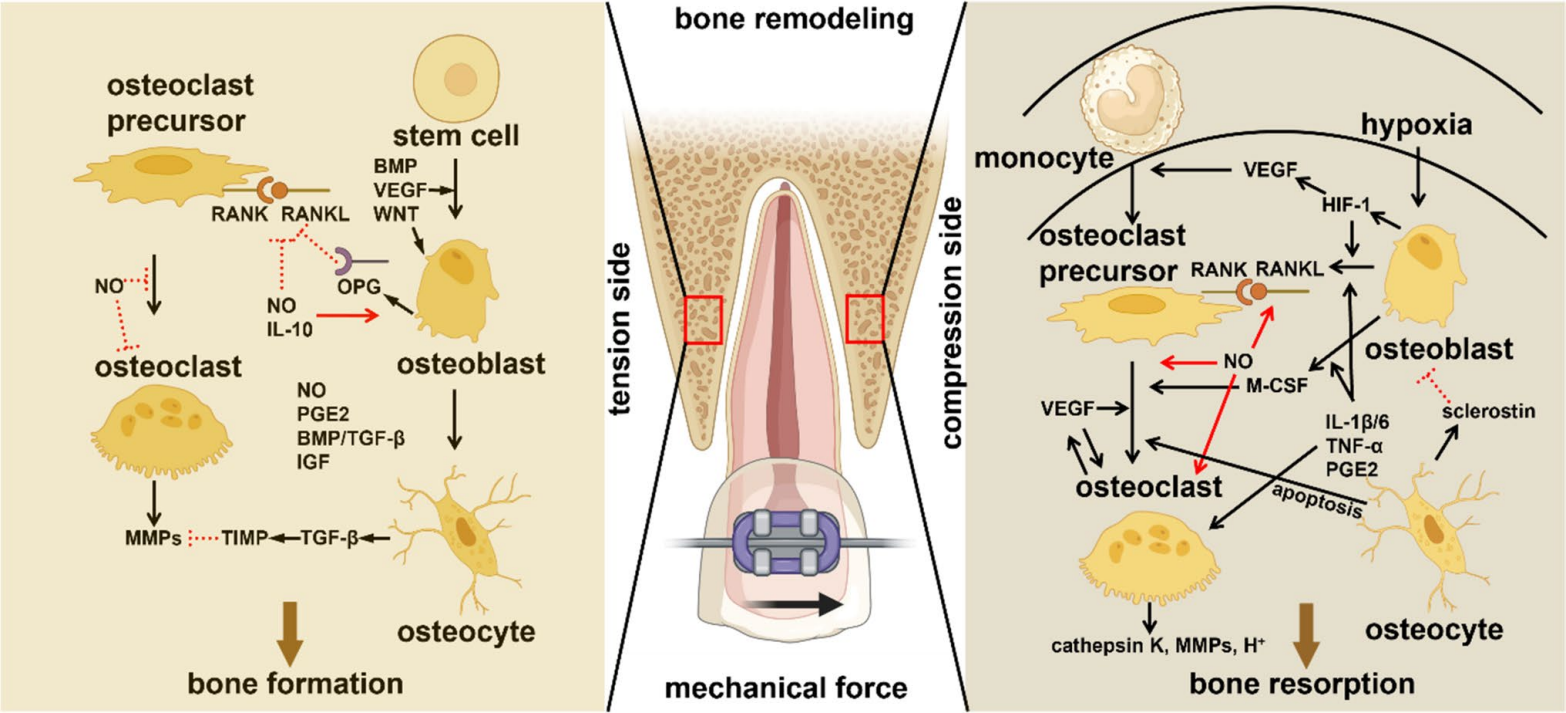
Schematic diagram of bone remodeling at the “compression side” and “tension side” during orthodontic tooth movement. Mechanical force is applied to the teeth. Bone remodeling starts at the “compression side” by means of osteoclastic bone resorption regulated by several (bio)chemical factors, e.g. NO, PGE_2_, sclerostin, TNF-α, IL-1β/6, RANK/RANKL, M-CSF, VEGF, cathepsin K, and MMPs. At the “tension side” osteoblastic bone formation is also regulated by (bio)chemical factors, e.g. NO, PGE_2_, IL-10, RANK/RANKL, BMP, VEGF, OPG, IGF, TGF-β, TIMP, and MMPs. NO, nitric oxide; PGE_2_, prostaglandin E_2_; TNF-α, tumor necrosis factor-α; IL-1β/6, interleukin-1β/6; RANK, receptor activator of nuclear-κB; RANKL, receptor activator of nuclear factor- κB ligand; M-CSF, macrophage-colony stimulating factor; VEGF, vascular endothelial growth factor; OPG, osteoprotegerin; IGF, insulin-like growth factor; TGF-β, transforming growth factor-β; TIMP, tissue inhibitor of metalloproteinases; MMPs, matrix metalloproteinases

Bone is able to adapt its mass and structure to mechanical loading [17]. Enhanced mechanical loading on bone in vivo leads to increased bone mass and mineral density [18, 19•], while decreased mechanical loading on bone lowers bone mass, mineral content, and bone matrix protein production [20, 21]. Bone adaptation occurs during bone remodeling [22]. This process of functional bone adaptation serves to acquire bones that unite a suitable resistance against mechanical failure with a minimum use of material [23, 24]. Clinically relevant functional bone adaptation occurs for instance during orthodontic tooth movement. After orthodontic load application, the recruitment of osteoclasts leads to bone resorption at the site clinically referred to as “compression side”, whilst the recruitment of osteoblasts leads to bone formation at the site clinically referred to as “tension side” [25••] (Fig. 1). To better understand bone adaptation during orthontic tooth movement at the macroscopic level, more information regarding the role of ostocyte mechanotransduction is needed. Optimal tissue remodeling facilitating tooth movement to other more desirable locations in the dental arch requires knowledge of how mechanical loading is converted into biochemical signals, identification of the cells and the signaling pathways involved, and the response mechanisms activated. We postulate that osteocyte mechanotransduction has a pivotal role in bone remodeling during orthodontic tooth movement, and consequently that orthodontic tooth movement may be improved rapidly and effectively when osteocytes are optimally mechanically loaded. Therefore, in this review, we focus on the role of osteocyte mechanotransduction, as well as on the modulating signaling molecules and signaling pathways involved in orthodontic tooth movement.

## Bone Homeostasis

Bone adaptation to mechanical loading plays a critical role in bone growth and maintenance of bone homeostasis. Bone matrix is continuously resorbed (degraded) by osteoclasts and rebuilt by osteoblasts at approximately 1–2 million microscopic sites in every adult skeleton [26]. Bone resorption carried out by osteoclasts in a newly formed osteon takes around 3 weeks, whereas building by osteoblasts takes around 3–4 months [26]. In bone, osteoblast differentiation and function are affected by transcription factors, e.g., Cbfa1, growth factors and morphogens, like bone morphogenetic proteins (BMPs), fibroblast growth factors, Wnt, as well as their inhibitors, e.g. sclerostin [27]. Osteoclast differentiation and function are affected by different molecules, such as the cell surface receptor RANK (receptor activator of nuclear factor kappa B (NFκB)), RANK ligand (RANKL), cytokines such as macrophage-colony stimulating factor (M-CSF), and osteoprotegerin (OPG; decoy receptor for RANKL) [27]. In healthy adults, bone formation and resorption are balanced, thereby ensuring maintenance of bone strength. The balance between bone formation and resorption is affected by several factors, most notably ageing, hormones, and mechanical loading [28]. Several diseases are associated with a disbalance in bone formation and resorption, i.e. osteoporosis, Paget’s disease, several types of cancer (e.g., solid tumors, hematopoietic malignancies), and inflammatory diseases (e.g., rheumatoid arthritis) [26]. Overloading around badly designed (oral) implants also affects remodeling, and causes bone loss. However, bone remodeling can also be used e.g., during distraction osteogenesis of the mandible, or for orthodontic tooth movement [29, 30]. A disbalance in bone formation and resorption can cause bone loss resulting in fractures, which is painful and needs to be prevented. Therefore, it is crucial to maintain a remodeling balance.

## How do Teeth Move Orthodontically?

Orthodontic tooth movement comprises two major steps, i.e., osteoclastic resorption of existing bone at the “compression side”, and osteoblastic formation of new bone at the “tension side”. The coordinated cooperation of osteoclasts and osteoblasts during bone adaptation is generally believed to be orchestrated by osteocytes, which can respond to mechanical loading [13, 14]. Osteocytes respond to opposite flow patterns by stimulating osteoclast activity at the “compression side” with low flow, and by facilitating bone formation by osteoblasts around the “tension side” with high flow. Osteoclast and osteoblast activity are therefore related to opposite flow patterns, and the mechanosensitive osteocytes are of utmost importance to orchestrate the process of remodeling.

Lowering of normal strain of the periodontal ligament occurs at the “compression side” during orthodontic tooth movement. This leads to local stasis of extracellular fluid in the bone canalicular network. Increased strain in bone caused by traction of the periodontal ligament leads to increased fluid flow in the canaliculi at the “tension side” during orthodontic tooth movement. Periodontal ligament cells are stretched or compressed during orthodontic tooth movement [31], and cytokines such as tumor necrosis factor-α (TNF-α) and interleukin-1 (IL-1) are produced [32]. Along with TNF-α in the sulcus, osteocyte apoptosis is caused at the “compression side” by local stress shielding resulting from decreased functioning of the periodontal ligament [33]. This causes almost complete fluid stasis in the osteocyte canaliculi [34], absence of fluid shear stress on the osteocytes, and decreased nitric oxide (NO) production [33]. The insufficient NO production causes osteocytes to enter apoptosis via enhanced caspase-3 gene expression and activity, and decreased Bcl-2 gene expression [35]. Apoptotic osteocytes then attract osteoclasts [33, 36], leading to bone resorption and remodeling. Moreover, the absence of mechanical loading-induced NO production at the “compression side” results in the absence of inhibition of osteoclast activity, leading to bone resorption. Finally, pro-inflammatory cytokines activation of the inducible nitric oxide synthase (iNOS) pathway in osteocytes, and NO arisen from this pathway potentiates inflammation-induced bone resorption at the “compression side” [11, 37]. iNOS-positive osteocytes mainly appear in the compression area, whilst endothelial nitric oxide synthase (eNOS)-positive osteocytes are present in the tension area [38••]. This suggests that inflammation-induced bone resorption in the tension area is mediated by eNOS, en in the compression area by iNOS. It seems that both eNOS and iNOS are highly important regulators of bone remodeling during orthodontic force application [38••]. The mechanically meaningful attraction of osteoclasts towards the “compression side” and maintaining osteocyte viability at the “tension side”, explains osteoclast behaviour during orthodontic tooth movement, resulting in bone remodeling.

## Osteocyte Mechanotransduction

### Importance of Osteocytes in Bone

Osteocytes are former osteoblasts that have become entrapped in osteoid [12]. Osteocytes still produce matrix proteins, although osteoblast metabolic activity decreases after being encased in bone matrix [12]. Osteocytes have numerous (around 60 per cell) long slender cell processes containing microfilaments, that are organized during matrix formation before calcification. The entire bone matrix is penetrated by a network of canaliculi containing the cellular processes [39]. Osteocytes sense and respond to bone tissue strain, resulting in enhanced bone remodeling activity by osteoclasts at locations where bone remodeling is needed [12].

Osteocyte apoptosis affects bone remodeling [40]. It is associated with osteoclastic bone resorption in growing bone [41]. Enhanced osteocyte apoptosis is observed after estrogen withdrawal in rats [42], which suggests that estrogen loss during menopause compromises osteocyte viability, resulting in an altered response to mechanical loading and rapid bone loss. The local mechanisms inducing osteocyte apoptosis, and the local signals produced by apoptotic osteocytes to attract or target osteoclasts are largely unknown. Fluid shear stress protects osteocytes against apoptosis [43]. Apoptotic osteocytes likely represent a cellular signal for osteoclast recruitment leading to bone resorption [44•]. Osteoclasts have been shown to attack apoptotic osteocytes [45], indicating that osteocyte apoptosis regulates osteoclastic bone resorption during bone remodeling.

### Role of Osteocytes in Mechanotransduction

Osteocytes are the orchestrators of bone adaptation to mechanical loading [46]. They are embedded in bone, and contact neighbouring osteocytes via long slender cell processes running through canaliculi, which contain interstitial fluid [47]. These cell processes also contact osteoblasts, bone lining cells, and osteoclasts at the bone surface [48]. Throughout bone, this three-dimensional network of interconnected cells with its accompanying lacuna-canalicular porosity is mechanosensing site in bone [43]. How the osteocytes sense mechanical loading via this three-dimensional network is explained by the “canalicular flow hypothesis” [49]. When bone is mechanically loaded, the interstitial fluid is squeezed through the three-dimensional network, leading to a fluid flow [50]. This fluid flow leads to a strain-driven interstitial fluid movement through the canaliculi and along the osteocyte processes, which is sensed and transduced by the osteocytes [51]. This interstitial fluid flow stimulates signaling molecule production by osteocytes that stimulate osteoclastic bone resorption, or osteoblastic bone formation [14, 52]. Mechanotransduction then comprises the translation of canalicular flow by osteocytes into cell signals that recruit osteoclasts and osteoblasts [44•, 45]. Osteocyte-ablated mice are resistant to unloading-induced bone loss, providing evidence for a role of osteocyte mechanotransduction [53].

Orthodontic loading causes osteoclastic bone resorption at the “compression side” where normal strain of the periodontal ligament is decreased and local extracellular fluid stasis in the canaliculi occurs [54, 55]. It causes osteoblastic bone formation at the “tension side” where increased strain in bone caused by traction of the periodontal ligament leads to enhanced fluid flow in the canaliculi [2••]. Osteoclast and osteoblast activity are thus related to opposite flow patterns, while mechanosensitive osteocytes orchestrate bone remodeling [10, 11, 50]. Since local differences in the tensile strain magnitude and orientation exist in the jaw bone, osteocyte morphology and orientation differ locally [16•]. This reflect local differences in the osteocyte mechanosensitivity and bone quality, suggesting differences in dental implant success based on the location in the maxilla [16•].

### Signaling Molecules in Osteocyte Mechanotransduction

Mechanically loaded osteocytes produce signaling molecules like bone morphogenetic proteins (BMPs), Wnts, prostaglandins, osteopontin, sclerostin, NO, and RANKL, which modulate the recruitment, differentiation, and activity of osteoblasts and osteoclasts [14, 46, 56].

#### BMPs

BMPs are powerful osteoinductive proteins that belong to the transforming growth factor-β (TGF-β) superfamily [57]. The BMP-induced signaling pathway results in expression of osteogenic master transcription factors osterix, Runx2, and Dlx5 [58]. BMP-2 interacts with BMP-2 receptors, causing αvβ3 integrin activation and modulation of cell spreading and migration independent of matrix stiffness [59]. On the other hand, αvβ3 is required to inhibit glycogen synthase kinase-3β (GSK-3β) activity through the Src–FAK–ILK pathway [59]. BMP receptors and β3 integrin signaling converge to control focal adhesion dynamics and Smad signaling, coupling cell migration and fate commitment independent of matrix stiffness [59].

#### Wnts

Osteocytes produce anabolic molecules including Wnts during mechanical loading [60, 61]. Wnt-mediated canonical Wnt signaling pathway activation plays a role in tension force-induced bone remodeling during orthodontic tooth movement [62]. Mechanical loading enhances, but disuse decreases Wnt activity [63]. Wnt signaling pathways have different roles in skeletal homeostasis [64]. Canonical Wnt signaling begins when Wnt ligands bind to Frizzled and low-density lipoprotein receptor-related protein 5/6 (Lrp5/6) receptors on the cell membrane [65]. Wnt signaling activation facilitates β-catenin accumulation through inhibition of GSK-3β-induced β-catenin phosphorylation, followed by translocation of dephosphorylated β-catenin into the nucleus to induce LEF/TCF-responsive gene transcription [65]. β-catenin serves as one of the core hubs for mechanotransduction, and as a primary intracellular signal transducer for Wnt signaling pathways [66].

#### Prostaglandins

Periodontal ligament fibroblasts perform regulatory functions in the innate immune response, and respond to compressive and tensile forces during orthodontic treatment with prostaglandin release [67]. Prostaglandins are important effector molecules in the cellular responses to cytokines, especially IL-1, involved in inflammation caused by orthodontic tooth movement. Among the subclasses of prostaglandins, prostaglandin E_2_ (PGE_2_) at high concentrations is strongly related to bone resorption [68], inhibits osteoprotegerin, and stimulates the receptor activator of nuclear factor-κB RANKL, which causes increased cyclooxygenase-1 (COX-1) and cyclooxygenase-2 (COX-2) expression [69]. Acute disuse rapidly upregulates osteopontin by osteocytes, which likely mediates bone resorption, since osteopontin is a known osteoclast chemotaxant and modulator of osteoclast attachment to bone [70].

#### Osteopontin

Osteopontin is an integrin ligand in bone produced by osteoblasts and osteoclasts. Loss of osteopontin causes resistance to unloading-induced bone loss [71]. Osteopontin is a multifunctional protein, and is considered to be crucial for bone remodeling, biomineralization, and periodontal remodeling during orthodontic tooth movement [72]. It contributes to bone remodeling by promoting osteoclastogenesis and osteoclast activity through CD44 and αvβ3-mediated cell signaling [72]. Osteoblasts and osteoclasts are not changed in unloaded osteopontin knockout mice, which suggests that osteopontin is a prerequisite for enhanced osteoclastic bone resorption and decreased osteoblastic bone formation under disuse [73].

#### Sclerostin

Mechanical loading represses sclerostin through the histone deacetylases HDAC4 and HDAC5 in osteocytes [71, 74]. Moreover, fluid shear stress triggers FAK inactivation via dephosphorylation, thus driving the nuclear translocation of class IIa HDAC by inhibition of HDAC5 tyrosine 642 phosphorylation [71, 75]. Sclerostin (SOST) is a protein associated with the SOST gene, and is also produced by osteocytes [76]. Expression of sclerostin by osteocytes is intended to inhibit osteoblast activity and ultimately bone formation [77]. Mechanical loading has been shown to affect SOST gene expression, which leads to changes in bone remodeling [78].

#### NOS Expression and NO Production

Osteocytes significantly increase NO production as a result of mechanical stress, supporting the theory that osteocytes play a major role in orthodontic tooth movement. NO is a short-lived highly reactive free radical involved in e.g., the regulation of bone metabolism, and is produced through eNOS and/or iNOS activity [79]. Cultured osteocytes respond to fluid shear stress by enhanced NO production, and to a localized mechanical loading on the single osteocyte level [80, 81]. NO inhibits osteoclast activity [38••, 82], and mediates adaptive bone formation in vivo [79, 83]. NO is one of the key molecules involved in the regulation of bone homeostasis and activation or inhibition of osteoblasts and osteoclasts [53, 54]. NO promotes osteoblast differentiation and bone formation [84]. NO is strongly involved in the biomechanical response of the periodontium to orthodontic forces [85]. NO is expressed differently on the “tension side” and “compression side” during tooth movement, which explains its complex involvement in bone remodeling [38••]. NO production occurs through tension force during orthodontic force in periodontal ligament cells via eNOS [38••, 86]. Additionally, NO exerts complex effects on the activity of both osteoblasts and osteoclasts, and the spatiotemporal NO generation may determine its specific biological effect on bone remodeling [87]. Little is known on the role and impact of NO in orthodontic tooth movement [82]. Moreover, expression patterns of the NOS isoforms iNOS, eNOS, and nNOS in periodontal ligament and bone during orthodontic tooth movement in rats revealed that all NOS isoforms are involved in orthodontic tooth movement, and that they are differently expressed at tension and pressure sides [86]. nNOS appeared more involved in early orthodontic tooth movement [86]. NOS expression in osteocytes did not change, suggesting that periodontal ligament cells rather than osteocytes are the mechanosensors in early orthodontic tooth movement [86].

#### RANKL

Osteocytes are also a great source for RANKL, another important molecule produced during orthodontic tooth movement. RANKL is one of the crucial cytokines driving osteoclast formation during mechanical loading, and thus bone remodeling [32]. Mice with an osteocyte-specific RANKL-deficiency are resistant to orthodontic tooth movement due to inhibition of osteoclastogenesis in periodontal tissue [88]. Thus, osteocytes are theoretically capable of orchestrating bone adaptation during orthodontic tooth movement by produce signaling molecules.

On the basis of the findings of the present review, mice or rats models have been used mostly to study the role of osteocytes in orthodontic tooth movement. A summary of selected key articles on the role of osteocyte mechanotransduction in orthodontic tooth movement is presented in Table 1. These articles report that orthodontic force affects expression of important biological molecules by osteocytes in mice or rats, and provide evidence that osteocytes play an integral role in the process of orthodontic tooth movement. Osteocyte mechanotransduction allows communication with other osteocytes, osteoblasts, and osteoclasts. It converts mechanical loading during orthodontic tooth movement into biochemical responses that control bone mineralization [89]. Interestingly, the number of osteoblasts at the “tension side” of the periodontal ligament space is also significantly reduced due to a possible coupling mechanism in alveolar bone [88]. Matrix extracellular phosphoglycoprotein (MEPE) and dentin matrix protein 1 (DMP1) are involved in (de)mineralisation of the osteocyte microenvironment. The effect of mechanical loading on MEPE and DMP1 expression during orthodontic tooth movement in mice has been investigated [90]. The results showed different expression of MEPE and DMP-1 during early (day 1) and late (days 4–7) periods of mechanical loading [90].

**Table 1:** Key references on osteocyte mechanotransduction in orthodontic tooth movement in vivo. M, male; F, female; n.i., not indicated; Ocy, osteocytes; OC, osteoclasts; WT, wild type; Tg, transgenic; OTM, orthodontic tooth movement; #, number; ^+^, positive; PDL, periodontal ligament; Scl, sclerostin; eNOS, endothelial nitric oxide synthase; iNOS, inducible nitric oxide synthase; nNOS, neuronal nitric oxide synthase

Ref. #	Species, in vivo/ in vitro	Gender, M/F	Age, wk	Inter-vention	Outcome parameters	Significance
[91]	Mouse, (WT, Tg), in vivo	M	8	OTM	# Ablated OCY, # OC, eroded bone surface, lacunocanalicular system	In vivo demonstration of OCY involvement in osteoclastic bone resorption during OTM
[86]	Rat, In vivo	M	n.i	OTM	# iNOS^+^, nNOS^+^, eNOS^+^ cells	PDL cells respond to OTM by NO production; iNOS, nNOS, and eNOS are involved, with nNOS more involved in early OTM
[92]	Mouse, in vivo & in vitro	M	8	OTM	Scl expression, gene expression	PDL-derived Scl is principal mediator of bone remodeling through paracrine effect on OCY
[88]	Mouse (WT, Tg), in vivo	M	12	OTM	# OC, gene expression	Key role for OCY-derived RANKL in alveolar bone remodeling, establishing molecular basis for orthodontic force-mediated bone resorption
[93]	Rat, in vivo	M	46	OTM	# iNOS^+^, eNOS^+^ OCY	Key role for eNOS and iNOS in bone remodeling during OTM; iNOS^+^ OCY are present in compression area, and eNOS^+^ OCY in tension area

## Orthodontic Tooth Movement and Osteocyte Signaling Pathways

During orthodontic tooth movement, increased fluid flow and stretching of periodontal ligament fibers on the “tension side” are followed by stimulation of osteoblast activity leading to increased bone formation, while disruption of fluid flow and cell death due to lack of nutrition on the “compression side” leads to bone resorption and allows tooth movement [94]. In general three major components represent the response to mechanical loading during orthodontic treatment: 1) the osteocyte mechanoresponse, 2) osteoclastogenesis, and 3) osteogenesis [95••]. Osteoblast differentiation and function are affected by specific transcription factors, growth factors and morphogens, as well as their inhibitors, while osteoclast differentiation and function are affected by different molecules, such as RANKL, cytokines, and osteoprotegerin [96] (Fig. 1). Although the underlying biological and biomechanical processes of orthodontic tooth movement are still not fully elucidated, there are several studies showing that osteocytes play a major role in controlling the entire response to mechanical loading [97–99].

### Signaling Pathways Activated by Mechanical Loading

The two major signaling pathways activated by mechanical loading are the Wnt/β-catenin pathway and the RANKL pathway [100].

#### Wnt/β-catenin Pathway

The Wnt signaling pathway is one of the key regulators of bone metabolism identified in mechanoresponsive osteocytes [101]. The Wnt-sensitive signaling pathway is separated into the canonical signaling pathway which depends on the function of β-catenin (the so-called Wnt/β-catenin pathway) and the non-canonical pathway [65]. The canonical signaling pathway includes critical molecular cascades for cellular metabolism [102]. It is essential for remodeling of bone/cementum in response to orthodontic force [103]. Wnt secretion by osteocytes during orthodontic tooth movement leads to the activation of β-catenin signaling and upregulation of genes involved in osteoblast differentiation and activity, resulting in bone formation [101]. In addition, modulation of the expression of the SOST gene encoding, which encodes sclerostin, an inhibitor of Wnt signaling, stimulates bone formation on the “tension side” and inhibits bone formation on the “compression side”, thereby regulating orthodontic tooth movement [104].

#### RANKL Pathway

The RANKL pathway defines bone mass as it discharges osteoclast progenitors into the circulation [105]. RANKL-induced osteoclast activation is vital to homeostasis over progenitor recruitment connecting bone remodelling with haematopoiesis regulation [105]. Sclerostin downregulates osteoblast activity and function [92]. It may ultimately upregulate RANKL gene expression and stimulate osteoclastogenesis during mechanical loading resulting in bone resorption, since the RANKL pathway is important for the regulation of bone resorption [106]. RANKL is produced by osteoblasts and osteocytes, and binds to its receptor RANK on the surface of osteoclasts [107]. This interaction activates a signaling cascade that leads to the differentiation, activation, and survival of osteoclasts, resulting in bone resorption [108]. During orthodontic tooth movement, RANKL expression increases in gingival crevicular fluid in juvenile patients [109]. RANKL accelerates orthodontic tooth movement by increasing the number of osteoclasts and bone resorption at the compression side [105].

### Other Pathways

Osteocytes also regulate the production of M-CSF, OPG, and other cytokines during orthodontic tooth movement (Fig. 1). Other signaling molecules or proteins might be involved in the process of osteocyte mechanotransduction during orthodontic tooth movement, including connexin-43, transient receptor potential vanilloid 4, piezo1, sphingosine-1-phosphate, zinc finger protein, extracellular signal regulated kinase, adenosine triphosphate, extracellular vesicles, and cyclic adenosine monophosphate [110]. Moreover, there is in vivo evidence for a key role of osteocytes in orthodontic tooth movement [88].

## Future Perspective

New concepts regarding the biological foundation of force-induced tooth movement will further improve the field of orthodontics. The orthodontic community now has tools for exploring the cellular and molecular events involved in orthodontic tooth movement, including the use of stem cells capable of differentiating into osteoblasts and osteoclasts. The periodontal ligament and alveolar bone represent a functional unit undergoing remodeling during orthodontic tooth movement. Complex molecular signaling is responsible for converting mechanical stresses into biochemical events, with a net result of bone apposition and/or bone resorption. Despite our improved understanding of mechanical and biochemical signaling mechanisms, it is still largely unknown how mechanical stresses regulate the differentiation of stem/progenitor cells into osteoblast and osteoclast lineages. A better understanding of the impact and role of osteocyte mechanotransduction during orthodontic tooth movement, as well as osteoblast differentiation from mesenchymal stem/progenitor cells and osteoclastogenesis from the hematopoietic/monocyte lineage, helps guide our efforts towards new approaches to solve current challenges of orthodontic treatment. In particular, it is assumed that osteocyte mechanotransduction plays a pivotal role in bone remodeling during orthodontic tooth movement, and consequently that orthodontic tooth movement may be improved effectively and rapidly if osteocytes are optimally mechanically loaded. Since the tensile strain magnitude and orientation is locally different in jaw bone, local differences in the osteocyte morphology and orientation exist. This reflect local differences in the osteocyte mechanosensitivity and bone quality, suggesting differences in dental tooth movement success based on the location in the maxilla. A view on the relationship between tensile strain and the osteocyte morphology and orientation in the maxillary bone might contribute to a better understanding of the cellular processes that lead to different bone qualities in various dental tooth movement positions and, eventually, to the success of dental tooth movement in the maxilla. A mechanistic understanding of the optimal force level resulting in optimal mechanical loading on the periodontal ligament for orthodontic tooth movement could help in developing acceleratory techniques for orthodontics. Future research is needed to unravel the underlying biological and biomechanical processes leading to optimal mechanical loading on the periodontal tissues during orthodontic tooth movement, and enable the translation of biological concepts into clinical practice.

## Conclusion

In this review we attempt to shed light on the impact and role of osteocyte mechanotransduction during orthodontic tooth movement. Osteocyte mechanotransduction plays a significant role in bone remodeling during orthodontic tooth movement. Osteocytes use paracrine signaling, stimulation and secretion of different cytokines, hormones, and enzymes to interact with other bone cells, e.g., osteoblasts and osteoclasts, to maintain a delicate balance between bone formation and resorption. It has also been demonstrated that NOS, RANKL, sclerostin, and many other essential molecules are expressed in response to mechanical loading through osteocyte mechanotransduction. For various reasons, mostly animal studies and only few human studies have been performed to demonstrate the important regulatory role of osteocytes in orthodontic tooth movement. To fully comprehend the role of osteocyte mechanotransduction in orthodontic tooth movement, further studies are required unraveling the biological pathways involved in order to gain more insight into how to optimize orthodontic treatment in humans and enhance patient outcomes.

## References

[1] McCormack SW, Witzel U, Watson PJ, Fagan MJ, Gröning F (2014). The biomechanical function of periodontal ligament fibres in orthodontic tooth movement. PLoS ONE.

[2] Li Y, Zhan Q, Bao M, Yi J, Li Y (2021). Biomechanical and biological responses of periodontium in orthodontic tooth movement: up-date in a new decade. Int J Oral Sci.

[3] Cuoghi OA, Tondelli PM, Mendonça MR, Aiello CA, Da Costa SC, Tanaka OM (2018). Effect of different types of force on the amount of tooth movement, hyaline areas, and root resorption in rats. Eur J Gen Dent.

[4] Haas AN, Pannuti CM, Andrade AKP, Escobar EC, Almeida ER, Costa FO, Cortelli JR, Cortelli SC, Rode SD, Pedrazzi V (2014). Mouthwashes for the control of supragingival biofilm and gingivitis in orthodontic patients: Evidence-based recommendations for clinicians. Braz Oral Res.

[5] Heymann GC, Tulloch JFC (2006). Implantable devices as orthodontic anchorage: A review of current treatment modalities. J Esthet Restor Dent.

[6] Erbe C, Heger S, Kasaj A, Berres M, Wehrbein H (2022). Orthodontic treatment in periodontally compromised patients: A systematic review. Clin Oral Investig.

[7] Bhardwaj A, Kumar Sharma A, Mishra K, Jeswani R (2020). Skeletal anchorage system [miniplates] - An orthodontic perspective - A review. Acta Sci Dent Sci.

[8] Ali MJ, Bhardwaj A, Khan MS, Alwadei F, Gufran K, Alqahtani AS, Alqhtani NR, Alasqah M, Alsakr AM, Alghabban RO (2022). Evaluation of stress distribution of maxillary anterior egment during en masse retraction using posterior mini screw: A finite element study. Appl Sci.

[9] Pereira Alexandre L, Nava Lopes Cançado L, Renato Jordão C (2019). Absolute orthodontic anchorage: A brief review. Int J Appl Dent Sci..

[10] Schätzle M, Männchen R, Zwahlen M, Lang NP (2009). Survival and failure rates of orthodontic temporary anchorage devices: A systematic review. Clin Oral Implants Res.

[11] Li Y, Jacox LA, Little SH, Ko CC (2018). Orthodontic tooth movement: The biology and clinical implications. Kaohsiung J Med Sci.

[12] Hadjidakis DJ, Androulakis II (2006). Bone remodeling. Ann NY Acad Sci..

[13] Bouchard AL, Dsouza C, Julien C, Rummler M, Gaumond M-H, Cermakian N, Willie BM (2022). Bone adaptation to mechanical loading in mice is affected by circadian rhythms. Bone.

[14] Klein-Nulend J, Bakker AD, Bacabac RG, Vatsa A, Weinbaum S (2013). Mechanosensation and transduction in osteocytes. Bone.

[15] Bacabac RG, Mizuno D, Schmidt CF, MacKintosh FC, Van Loon JJWA, Klein-Nulend J, Smit TH (2008). Round versus flat: Bone cell morphology, elasticity, and mechanosensing. J Biomech.

[16] Wu V, van Oers RFM, Schulten EAJM, Helder MN, Bacabac RG, Klein-Nulend J (2018). Osteocyte morphology and orientation in relation to strain in the jaw bone. Int J Oral Sci.

[17] Turner CH (1998). Three rules for bone adaptation to mechanical stimuli. Bone.

[18] Kalyanaraman H, Pal China S, Cabriales JA, Moininazeri J, Casteel DE, Garcia JJ, Wong VW, Chen A, Sah RL, Boss GR (2023). Protein kinase G2 is essential for skeletal homeostasis and adaptation to mechanical loading in male but not female mice. J Bone Miner Res.

[19] Haxhi J, Mattia L, Vitale M, Pisarro M, Conti F, Pugliese G (2022). Effects of physical activity/exercise on bone metabolism, bone mineral density and fragility fractures. Int J Bone Fragility.

[20] Kumar G, Narayan B. Regulation of bone formation by applied dynamic loads. In Classic Papers in Orthopaedics; Springer London: London, 2014; pp. 511–513.

[21] Gabel L, Liphardt A-M, Hulme PA, Heer M, Zwart SR, Sibonga JD, Smith SM, Boyd SK (2022). Pre-flight exercise and bone metabolism predict unloading-induced bone loss due to spaceflight. Br J Sports Med.

[22] Kameo Y, Miya Y, Hayashi M, Nakashima T, Adachi T (2020). In silico experiments of bone remodeling explore metabolic diseases and their drug treatment. Sci Adv.

[23] Robling AG, Castillo AB, Turner CH (2006). Biomechanical and molecular regulation of bone remodeling. Annu Rev Biomed Eng.

[24] Bauer TW, Muschler GF (2000). Bone graft materials. Clin Orthop Relat Res.

[25] Kirschneck C, Bauer M, Gubernator J, Proff P, Schröder A (2020). Comparative assessment of mouse models for experimental orthodontic tooth movement. Sci Rep.

[26] Rodan GA, Martin TJ (2000). Therapeutic approaches to bone diseases. Science.

[27] Tsourdi E, Jähn K, Rauner M, Busse B, Bonewald LF (2018). Physiological and pathological osteocytic osteolysis. J Musculoskelet Neuronal Interact.

[28] Robling AG, Castillo AB, Turner CH (2006). Biomechanical and molecular regulation of bone remodeling. Annu Rev Biomed Eng.

[29] Altay B, Dede EÇ, Özgul Ö, Atıl F, Koçyiğit İD, Orhan K, Tekin U, Korkusuz P, Önder ME (2020). Effect of systemic oxytocin administration on new bone formation and distraction rate in rabbit mandible. J Oral Maxillofac Surg.

[30] Pereira LJ, Macari S, Coimbra CC, Pereira TDSF, Barrioni BR, Gomez RS, Silva TA, Paiva SM (2020). Aerobic and resistance training improve alveolar bone quality and interferes with bone-remodeling during orthodontic tooth movement in mice. Bone.

[31] Zhang X, Chen D, Zheng J, Deng L, Chen Z, Ling J, Wu L (2019). Effect of microRNA-21 on hypoxia-inducible factor-1α in orthodontic tooth movement and human periodontal ligament cells under hypoxia. Exp Ther Med.

[32] Marahleh A, Kitaura H, Ohori F, Noguchi T, Nara Y, Pramusita A, Kinjo R, Ma J, Kanou K, Mizoguchi I (2021). Effect of TNF-α on osteocyte RANKL expression during orthodontic tooth movement. J Dent Sci.

[33] Tan SD, Kuijpers-Jagtman AM, Semeins CM, Bronckers ALJJ, Maltha JC, Von Den Hoff JW, Everts V, Klein-Nulend J (2006). Fluid shear stress inhibits TNFα-induced osteocyte apoptosis. J Dent Res.

[34] Tan SD, de Vries TJ, Kuijpers-Jagtman AM, Semeins CM, Everts V, Klein-Nulend J (2007). Osteocytes subjected to fluid flow inhibit osteoclast formation and bone resorption. Bone.

[35] Bakker A, Klein-Nulend J, Burger E (2004). Shear stress inhibits while disuse promotes osteocyte apoptosis. Biochem Biophys Res Commun.

[36] Wang X, He Y, Tian S, Zhu F, Huang B, Zhang J, Chen Z, Wang H (2019). Fluid shear stress increases osteocyte and inhibits osteoclasts via downregulating receptor-activator of nuclear factor kb (RANK)/osteoprotegerin expression in myeloma microenvironment. Med Sci Monit.

[37] Van’t Hof RJ, Ralston SH (2001). Nitric oxide and bone. Immunology.

[38] Yan T, Xie Y, He H, Fan W, Huang F (2021). Role of nitric oxide in orthodontic tooth movement. Int J Mol Med.

[39] Wasserman E. Differentially load-regulated gene expression in mouse trabecular osteocytes, Dissertation ETH Zurich, No. 18938, 2010.

[40] Bolamperti S, Villa I, Rubinacci A (2022). Bone remodeling: an operational process ensuring survival and bone mechanical competence. Bone Res.

[41] Ma J, Wang A, Zhang H, Liu B, Geng Y, Xu Y, Zuo G, Jia P (2022). Iron overload induced osteocytes apoptosis and led to bone loss in Hepcidin−/− mice through increasing sclerostin and RANKL/OPG. Bone.

[42] McNamara LM (2021). Osteocytes and estrogen deficiency. Curr Osteoporos Rep.

[43] Iandolo D, Strigini M, Guignandon A, Vico L (2021). Osteocytes and weightlessness. Curr Osteoporos Rep.

[44] Marahleh A, Kitaura H, Ohori F, Noguchi T, Mizoguchi I (2023). The osteocyte and its osteoclastogenic potential. Front Endocrinol.

[45] Klein-Nulend J, Nijweide PJ, Burger EH (2003). Osteocyte and bone structure. Curr Osteoporos Rep.

[46] Klein-Nulend J, Bacabac RG, Bakker AD (2012). Mechanical loading and how it affects bone cells: The role of the osteocyte cytoskeleton in maintaining our skeleton. Eur Cells Mater.

[47] Bakker AD, Klein-Nulend J, Burger EH (2003). Mechanotransduction in bone cells proceeds via activation of COX-2, but not COX-1. Biochem Biophys Res Commun.

[48] Zappalà A, Romano IR, D’Angeli F, Musumeci G, Lo Furno D, Giuffrida R, Mannino G (2023). Functional roles of connexins and gap junctions in osteo-hondral cellular components. Int J Mol Sci.

[49] Jin J, Bakker AD, Wu G, Klein-Nulend J, Jaspers RT (2019). Physicochemical niche conditions and mechanosensing by osteocytes and myocytes. Curr Osteoporos Rep.

[50] van Tol AF, Roschger A, Repp F, Chen J, Roschger P, Berzlanovich A, Gruber GM, Fratzl P, Weinkamer R (2020). Network architecture strongly influences the fluid flow pattern through the lacunocanalicular network in human osteons. Biomech Model Mechanobiol.

[51] Wang H, Du T, Li R, Main RP, Yang H (2022). Interactive effects of various loading parameters on the fluid dynamics within the lacunar-canalicular system for a single osteocyte. Bone.

[52] Zheng L, Zhou D, Ju F, Liu Z, Yan C, Dong Z, Chen S, Deng L, Chan S, Deng J (2023). Oscillating fluid flow activated osteocyte lysate-based hydrogel for egulating osteoblast/osteoclast homeostasis to enhance bone repair. Adv Sci.

[53] Tatsumi S, Ishii K, Amizuka N, Li M, Kobayashi T, Kohno K, Ito M, Takeshita S, Ikeda K (2007). Targeted ablation of osteocytes induces osteoporosis with defective mechanotransduction. Cell Metab.

[54] Sun C, Rankovic MJ, Folwaczny M, Stocker T, Otto S, Wichelhaus A, Baumert U (2022). Effect of different parameters of in vitro static tensile strain on human periodontal ligament cells simulating the tension side of orthodontic tooth movement. Int J Mol Sci.

[55] Roth CE, Craveiro RB, Niederau C, Malyaran H, Neuss S, Jankowski J, Wolf M (2022). Mechanical compression by simulating orthodontic tooth Movement in an in vitro model modulates phosphorylation of AKT and MAPKs via TLR4 in human periodontal ligament cells. Int J Mol Sci.

[56] Cao W, Helder MN, Bravenboer N, Wu G, Jin J, ten Bruggenkate CM, Klein-Nulend J, Schulten EAJM (2020). Is there a governing role of osteocytes in bone tissue regeneration?. Curr Osteoporos Rep.

[57] Centrella M, Horowitz MC, Wozney JM, Mccarthy TL (1994). Transforming growth factor-β gene family members and bone. Endocr Rev.

[58] Lee MH, Kwon TG, Park HS, Wozney JM, Ryoo HM (2003). BMP-2-induced Osterix expression is mediated by Dlx5 but is independent of Runx2. Biochem Biophys Res Commun.

[59] Fourel L, Valat A, Faurobert E, Guillot R, Bourrin-Reynard I, Ren K, Lafanechère L, Planus E, Picart C, Albiges-Rizo C (2016). β3 integrin-mediated spreading induced by matrix-bound BMP-2 controls Smad signaling in a stiffness-independent manner. J Cell Biol.

[60] Ei Hsu Hlaing E, Ishihara Y, Odagaki N, Wang Z, Ikegame M, Kamioka H (2020). The expression and regulation of Wnt1 in tooth movement–initiated mechanotransduction. Am J Orthod Dentofac Orthop..

[61] Santos A, Bakker AD, Zandieh-Doulabi B, Semeins CM, Klein-Nulend J (2009). Pulsating fluid flow modulates gene expression of proteins involved in Wnt signaling pathways in osteocytes. J Orthop Res.

[62] Fu HD, Wang BK, Wan ZQ, Lin H, Chang ML, Han GL (2016). Wnt5a mediated canonical Wnt signaling pathway activation in orthodontic tooth movement: Possible role in the tension force-induced bone formation. J Mol Histol.

[63] Santos A, Bakker AD, Zandieh-Doulabi B, de Blieck-Hogervorst JMA, Klein-Nulend J (2010). Early activation of the β-catenin pathway in osteocytes is mediated by nitric oxide, phosphatidyl inositol-3 kinase/Akt, and focal adhesion kinase. Biochem Biophys Res Commun.

[64] Baron R, Kneissel M (2013). WNT signaling in bone homeostasis and disease: from human mutations to treatments. Nat Med.

[65] Clevers H, Nusse R (2012). Wnt/β-catenin signaling and disease. Cell.

[66] Uzer G, Bas G, Sen B, Xie Z, Birks S, Olcum M, McGrath C, Styner M, Rubin J (2018). Sun-mediated mechanical LINC between nucleus and cytoskeleton regulates βcatenin nuclear access. J Biomech.

[67] Schröder A, Küchler EC, Omori M, Spanier G, Proff P, Kirschneck C (2019). Effects of ethanol on human periodontal ligament fibroblasts subjected to static compressive force. Alcohol.

[68] Jose JA, Somaiah S, Muddaiah S, Shetty B, Reddy G, Roopa S (2018). A comparative evaluation of interleukin 1 beta and prostaglandin E2 with and without low-level laser therapy during en masse retraction. Contemp Clin Dent.

[69] Vansant L, Cadenas De Llano-Pérula M, Verdonck A, Willems G (2018). Expression of biological mediators during orthodontic tooth movement: A systematic review. Arch Oral Biol..

[70] Luukkonen J, Hilli M, Nakamura M, Ritamo I, Valmu L, Kauppinen K, Tuukkanen J, Lehenkari P (2019). Osteoclasts secrete osteopontin into resorption lacunae during bone resorption. Histochem Cell Biol.

[71] Wang L, You X, Zhang L, Zhang C, Zou W (2022). Mechanical regulation of bone remodeling. Bone Res.

[72] Singh A, Gill G, Kaur H, Amhmed M, Jakhu H (2018). Role of osteopontin in bone remodeling and orthodontic tooth movement: a review. Prog Orthod.

[73] Ishijima M, Rittling SR, Yamashita T, Tsuji K, Kurosawa H, Nifuji A, Denhardt DT, Noda M (2002). Enhancement of osteoclastic bone resorption and suppression of osteoblastic bone formation in response to reduced mechanical stress do not occur in the absence of osteopontin. J Exp Med.

[74] Wein MN, Spatz J, Nishimori S, Doench J, Root D, Babij P, Nagano K, Baron R, Brooks D, Bouxsein M (2015). HDAC5 controls MEF2C-driven sclerostin expression in osteocytes. J Bone Miner Res.

[75] Sato T, Verma S, Andrade CDC, Omeara M, Campbell N, Wang JS, Cetinbas M, Lang A, Ausk BJ, Brooks DJ (2020). A FAK/HDAC5 signaling axis controls osteocyte mechanotransduction. Nat Commun.

[76] Omran A, Atanasova D, Landgren F, Magnusson P (2022). Sclerostin: From molecule to clinical biomarker. Int J Mol Sci.

[77] Poole KES, Van Bezooijen RL, Loveridge N, Hamersma H, Papapoulos SE, Löwik CW, Reeve J (2005). Sclerostin is a delayed secreted product of osteocytes that inhibits bone formation. FASEB J.

[78] Robling AG, Turner CH (2009). Mechanical signaling for bone modeling and remodeling. Crit Rev Eukaryot Gene Expr.

[79] Klein-Nulend J, van Oers RFM, Bakker AD, Bacabac RG (2014). Nitric oxide signaling in mechanical adaptation of bone. Osteoporos Int.

[80] Klein-Nulend J, Semeins CM, Ajubi NE, Nijweide PJ, Burger EH (1995). Pulsating fluid flow increases nitric oxide (NO) synthesis by osteocytes but not periosteal fibroblasts - correlation with prostaglandin upregulation. Biochem Biophys Res Commun.

[81] Vatsa A, Mizuno D, Smit TH, Schmidt CF, MacKintosh FC, Klein-Nulend J (2006). Bio imaging of intracellular NO production in single bone cells after mechanical stimulation. J Bone Miner Res.

[82] Hayashi K, Igarashi K, Miyoshi K, Shinoda H, Mitani H (2002). Involvement of nitric oxide in orthodontic tooth movement in rats. Am J Orthod Dentofac Orthop.

[83] Jeddi S, Yousefzadeh N, Kashfi K, Ghasemi A (2022). Role of nitric oxide in type 1 diabetes-induced osteoporosis. Biochem Pharmacol.

[84] Jin Z, Kho J, Dawson B, Jiang M-M, Chen Y, Ali S, Burrage LC, Grover M, Palmer DJ, Turner DL (2021). Nitric oxide modulates bone anabolism through regulation of osteoblast glycolysis and differentiation. J Clin Invest.

[85] Shirazi M, Nilforoushan D, Alghasi H, Dehpour AR (2002). The role of nitric oxide in orthodontic tooth movement in rats. Angle Orthod.

[86] Nilforoushan D, Manolson MF (2009). Expression of nitric oxide synthases in orthodontic tooth movement. Angle Orthod.

[87] Eriksen EF (2010). Cellular mechanisms of bone remodeling. Rev Endocr Metab Disord.

[88] Shoji-Matsunaga A, Ono T, Hayashi M, Takayanagi H, Moriyama K, Nakashima T (2017). Osteocyte regulation of orthodontic force-mediated tooth movement via RANKL expression. Sci Rep.

[89] Lerner UH (2012). Osteoblasts, osteoclasts, and osteocytes: Unveiling their intimate-associated responses to applied orthodontic forces. Semin Orthod.

[90] Gluhak-Heinrich J, Pavlin D, Yang W, MacDougall M, Harris SE (2007). MEPE expression in osteocytes during orthodontic tooth movement. Arch Oral Biol.

[91] Matsumoto T, Iimura T, Ogura K, Moriyama K, Yamaguchi A (2013). The role of osteocytes in bone resorption during orthodontic tooth movement. J Dent Res.

[92] Odagaki N, Ishihara Y, Wang Z, Ei Hsu Hlaing E, Nakamura M, Hoshijima M, Hayano S, Kawanabe N, Kamioka H (2018). Role of osteocyte-PDL crosstalk in tooth movement via SOST/sclerostin. J Dent Res..

[93] Tan SD, Xie R, Klein-Nulend J, Van Rheden RE, Bronckers ALJJ, Kuijpers-Jagtman AM, Von Den Hoff JW, Maltha JC (2009). Orthodontic force stimulates eNOS and iNOS in rat osteocytes. J Dent Res.

[94] Krishnan V, Davidovitch Z (2009). On a path to unfolding the biological mechanisms of orthodontic tooth movement. J Dent Res.

[95] Jeon HH, Teixeira H, Tsai A (2021). Mechanistic insight into orthodontic tooth movement based on animal studies: A critical review. J Clin Med.

[96] Kitaura H, Marahleh A, Ohori F, Noguchi T, Shen W-R, Qi J, Nara Y, Pramusita A, Kinjo R, Mizoguchi I (2020). Osteocyte-related cytokines regulate osteoclast formation and bone resorption. Int J Mol Sci.

[97] Bumann EE, Frazier-Bowers SA (2017). A new cyte in orthodontics: Osteocytes in tooth movement. Orthod Craniofacial Res.

[98] Jeon HH, Kang J, Li JM, Kim D, Yuan G, Almer N, Liu M, Yang S (2022). The effect of IFT80 deficiency in osteocytes on orthodontic loading-induced and physiologic bone remodeling: In vivo study. Life.

[99] Yoshimatsu M, Kitaura H, Morita Y, Nakamura T, Ukai T (2022). Effects of anti-mouse RANKL antibody on orthodontic tooth movement in mice. J Dent Sci.

[100] Li Y, Ling J, Jiang Q (2021). Inflammasomes in alveolar bone loss. Front Immunol.

[101] Duan P, Bonewald LF (2016). The role of the wnt/β-catenin signaling pathway in formation and maintenance of bone and teeth. Int J Biochem Cell Biol.

[102] Moon RT, Bowerman B, Boutros M, Perrimon N (2002). The promise and perils of Wnt signaling through β-catenin. Science (80-.)..

[103] Robinson JA, Chatterjee-Kishore M, Yaworsky PJ, Cullen DM, Zhao W, Li C, Kharode Y, Sauter L, Babij P, Brown EL (2006). Wnt/β-catenin signaling is a normal physiological response to mechanical loading in bone. J Biol Chem.

[104] Bullock WA, Pavalko FM, Robling AG (2019). Osteocytes and mechanical loading: The Wnt connection. Orthod Craniofacial Res.

[105] Kumar IG, Raghunath N, Kiran HJ (2022). RANK-RANKL-OPG: A current trends in orthodontic tooth movement and its role in accelerated orthodontics. Int J Appl Dent Sci..

[106] Wang P, Tang C, Wu J, Yang Y, Yan Z, Liu X, Shao X, Zhai M, Gao J, Liang S (2019). Pulsed electromagnetic fields regulate osteocyte apoptosis, RANKL/OPG expression, and its control of osteoclastogenesis depending on the presence of primary cilia. J Cell Physiol.

[107] Udagawa N, Koide M, Nakamura M, Nakamichi Y, Yamashita T, Uehara S, Kobayashi Y, Furuya Y, Yasuda H, Fukuda C (2021). Osteoclast differentiation by RANKL and OPG signaling pathways. J Bone Miner Metab.

[108] Wei T, Shan Z, Wen X, Zhao N, Shen G (2022). Dynamic alternations of RANKL/OPG ratio expressed by cementocytes in response to orthodontic-induced external apical root resorption in a rat model. Mol Med Rep.

[109] Kanzaki H, Chiba M, Arai K, Takahashi I, Haruyama N, Nishimura M, Mitani H (2006). Local RANKL gene transfer to the periodontal tissue accelerates orthodontic tooth movement. Gene Ther.

[110] Li MCM, Chow SKH, Wong RMY, Qin L, Cheung WH (2021). The role of osteocytes-specific molecular mechanism in regulation of mechanotransduction – A systematic review. J Orthop Transl.

